# Editorial: Cellular and Molecular Basis in Parasitic Diseases Control: Research Trends

**DOI:** 10.3389/fcell.2022.897858

**Published:** 2022-04-13

**Authors:** Jianbing Mu, Jun Cao, Gaoqian Feng, Qingfeng Zhang

**Affiliations:** ^1^ Laboratory of Malaria and Vector Research, National Institute of Allergy and Infectious Diseases, National Institutes of Health, Rockville, MD, United States; ^2^ National Health Commission Key Laboratory of Parasitic Disease Control and Prevention, Jiangsu Provincial Key Laboratory on Parasite and Vector Control Technology, Jiangsu Institute of Parasitic Diseases, Wuxi, China; ^3^ Burnet Institute for Medical Research and Public Health, Melbourne, VIC, Australia; ^4^ Key Laboratory of Spine and Spinal Cord Injury Repair and Regeneration of Ministry of Education, Tongji Hospital, Shanghai, China; ^5^ Clinical Center for Brain and Spinal Cord Research, School of Medicine, Tongji University, Shanghai, China

**Keywords:** parasitic diseases control, genomic, epigenomic, whole-genome sequencing (WGS), glycogenes, RNA secondary structurome, host-parasite interactions

Significant progress in parasite disease control has been made recently by applying novel tools and methodologies developed in genomic, epigenomic, proteomics, and metabolomics research. The research topic aimed to introduce some of the key developments and challenges of the field, focusing on parasite-induced immune response, genetic and epigenetic regulation mechanisms, and potential therapeutics. This Research Topic comprises 41 papers, including three reviews and 38 original research articles, spans topics that cover parasite biology, host pathology, epidemiology, and prevention and control programs.

**FIGURE 1:**
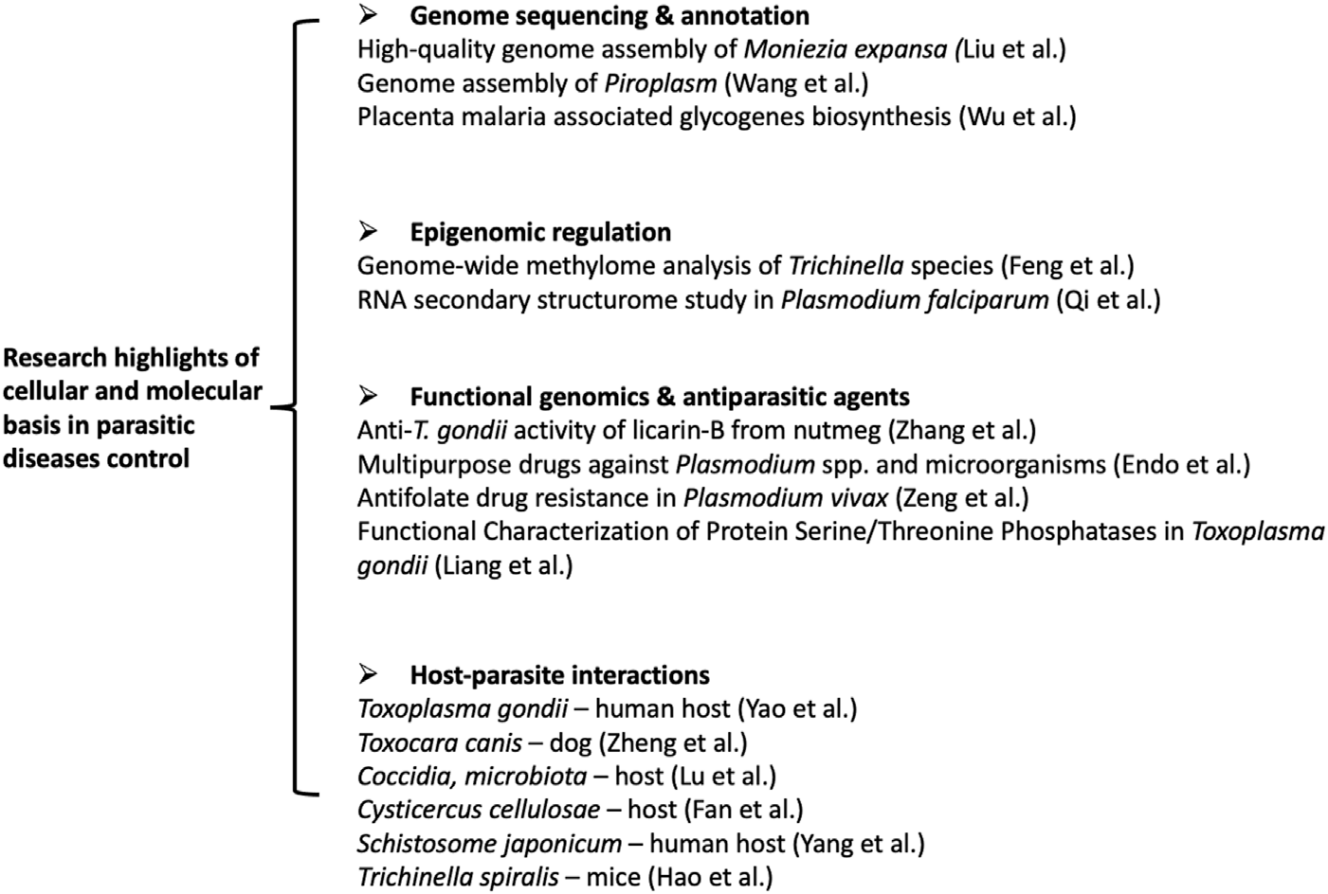
Seleted articles in the research topic of the cellular and molecular basis in parasitic dieases control-research trends.

Whole-genome sequencing (WGS) provides the most comprehensive characterization of the genome of a given organism. It allows for discovering previously unknown genes or variants associated with a unique parasite phenotype. Liu et al. successfully obtain a high-quality genome assembly of *Moniezia expansa (M. expansa*) by applying the next-generation sequencing techniques (Illumina, PacBio, and BioNano) (Figure 1). *De novo* sequencing and genome annotation reveal a total length of 142 Mb and 8,104 coding genes in the *M. expansa* genome. Notably, identifying this parasite’s specific fatty acid metabolism and reproductive stem cell regulatory network provides potential target molecules for effectively treating parasitic diseases. Although the long reads sequencing technology has dramatically changed the landscape of whole-genome sequencing, genome assembly is still a challenging task, particularly for the non-model species (Chen et al., 2021). An original article by Wang et al. systematically evaluated the performance of nine *de novo* assemblers for Oxford Nanopore Technology (ONT) on different coverage depth datasets of *Piroplasm* (Figure 1). The authors carefully provide the guidelines for selecting the assembly tools under specific conditions. The advantage and disadvantages of each tool are presented, which may provide critical information for improving the current *de novo* genome assembly tools and help for the future development of highly efficient assemblers. In addition to these *de novo* genome assembly studies, mining the well-annotated genome data with cross-species knowledge could likewise contribute to the novel molecular discovery, as shown by the study of Wu et al. in this research topic (Figure 1). It has long been recognized that expression of VAR2CSA on plasmodium parasite-infected RBC bind to distinct 4-O-sulfated chondroitin sulfate (CS) in the normal placenta (Ma et al., 2021). The parasite-specific molecular VAR2CSA was recently identified to bind various cancer cells through the same ligand (Salanti et al., 2015). However, the biosynthesis and functions of these chondroitin sulfates in tumors or placenta have not been fully elucidated. Here, Wu et al. systematically analyzed the key glycogenes in the biosynthesis of the common tetrasaccharide linkage, repeating disaccharide region of CS, and the glycogenes involved in sulfate modification in colorectal cancer and placenta. The results indicate that glycogenes in oncofetal chondroitin sulfate (ofCS) biosynthesis are differently expressed and correlated significantly with immune response in the placenta and colorectal cancer, indicating a potential biomarker or immune therapy targets for CRC.

In addition to the genomic study, epigenomics deals with global analyses of epigenetic changes across the entire genome, revealing how the genome is folded inside the cell’s nucleus, bound by proteins, and modified by enzymes, which regulate the gene expression without the DNA alteration. Feng et al. carried out the first comprehensive comparative epigenomic analysis in *Trichinella* species (Figure 1). The genome-wide methylome and transcriptome analysis of the 12 *Trichinella* species enables deciphering the environmental effects on differential adaptation and parasitism of *Trichinella*. Similarly, without the changes in its sequence, RNA molecules can promote rapid responses to changing environmental conditions and enhance regulation at the transcription level through structural transformation (Bevilacqua et al., 2016). The RNA secondary structurome study by Qi et al. revealed distinct thermoregulation in *plasmodium falciparum* (Figure 1). In this study, the authors applied *in vitro* and *in vivo* transcriptomic-wide secondary structurome approach, icSHAPE, to measure the temperature associated RNA structure changes in different stages of *P. falciparum*. Multi-omics analysis of transcriptome and RNA structure data reveals the specific RNA secondary structure in the transcriptional regulation for parasites in response to temperature changes. Identifying potential cold response molecules may yield new insights into the molecular mechanism of the parasite virulence and development.

Host-parasite interactions play a central role in the evolution and dramatically influence their biology. Identification of the critical determinants of the host-parasite interactions could provide novel molecular targets for developing tools against parasitic diseases. This research topic covered various interactions between parasites and their host (Figure 1), such as *Toxoplasma gondii*—human host (Yao et al.); *Toxocara canis*—dog (Zheng et al.); *Coccidia microbiota*—host (Lu et al.); *Cysticercus cellulosae*—host (Fan et al.); *Schistosome japonicum*—human host (Yang et al.) and *Trichinella spiralis*—mice (Hao et al.). For example, Yao et al. found that *T. gondii* virulence factor ROP18 of the type I RH strain (TgROP18I) interacted with human TRIM21. This E3 ligase plays a vital role in anti-infection responses against intracellular pathogens for its immune escape. The supporting evidence includes 1) the interaction of TgROP18I with human TRIM21, 2) the phosphorylation of TRIM21 promoted by gROP18I, 3) TgROP18I promoted TRIM21 degradation, and 4) TRIM21 restricted *T. gondii* replication through NF-κB activation. This study revealed the possible mechanisms of the different disease outcomes caused by type I and type III *T. gondii*. In the review article by Lu et al., *Coccidia microbiota* interactions and their effects on the host were discussed. The direct and indirect interactions between *Coccidia* and gut microbiota were highlighted. The role of mechanical mucosal damage, chemical mucosal damage, disruption of the immune system, and probiotics on *Coccidia* development were discussed.

Together, this research topic brings important new data and provides a broad and diverse overview of cellular and molecular basis in parasitic diseases control. The collected articles are timely and address important questions in the field with overwhelmingly positive feedback (total 534,000 views and over 1,000 views for most articles). We hope this research topic will promote further investigations and drive the field toward better control for parasitic diseases.
